# The application of ascorbic acid as a therapeutic feed additive to boost immunity and antioxidant activity of poultry in heat stress environment

**DOI:** 10.14202/vetworld.2022.685-693

**Published:** 2022-03-24

**Authors:** Truong Van Hieu, Budi Guntoro, Nguyen Hoang Qui, Nguyen Thi Kim Quyen, Farid Akbar Al Hafiz

**Affiliations:** 1 Department of Animal Science and Veterinary Medicine, School of Agriculture and Aquaculture, Tra Vinh University, Tra Vinh City, Vietnam; 2 Department of Livestock Social-Economics, Faculty of Animal Science, Universitas Gadjah Mada, Yogyakarta City, Indonesia

**Keywords:** antioxidant, ascorbic acid, immune system, poultry production

## Abstract

Ascorbic acid, widely known as vtamin C, is an essential nutrient for animals such as poultry. Ascorbic acid in poultry feed improves animal health and thus increases the growth performance of birds. Ascorbic acid can be used in the form of synthetic products or can be naturally obtained from fruits and plants. It is soluble in water and can be easily administered in drinking water and the diet. Poultry can synthesize ascorbic acid in the body. However, the performance of the animals can be improved by adding ascorbic acid to their diet. In addition, ascorbic acid is called an antioxidant and an anti-inflammatory. This increases their resistance to disease during the transition season. Ascorbic acid supplementation positively affects the stress response, especially during the dry season in tropical countries. Furthermore, supplementing ascorbic acid in the poultry’s diet improves resistance to diseases, regulates stress, and helps in the body’s oxidation process. Ultimately, this enhances the laying rate, egg hatch performance, and higher poultry productivity. For layers at the end of the laying period, it helps increase the quality of the eggshell and reduces the proportion of broken eggs. Ascorbic acid has a strong relationship with other vitamins such as vitamin E and other substances such as zinc, safflower oil, folic acid, and a fibrous diet. This review aims to synthesize all the information of ascorbic acid in the poultry’s diet, thereby providing the general role of ascorbic acid for the poultry industry.

## Introduction

The poultry industry has advanced over the years. Although poultry’s productivity has improved, it is still relatively low. In addition, the epidemic situation in poultry production is complex, and the diseases are not fully controlled. Therefore, the cost of veterinary drugs is high, which reduces the efficiency of poultry production.

Ascorbic acid is called an antioxidant compound with a chemical formulation and the properties as shown in Table-1 [1]. It has antioxidant and anti-inflammatory effects. Therefore, it is effective for poultry in cases of inflammation, oxidative stress, and infection [2]. Ascorbic acid is not an essential nutrient for poultry as it can be synthesized from poultry through the biosynthetic pathway [3]. However, it is considered an essential nutrient in two cases: (1) It is important when birds do not synthesize enough ascorbic acid in the native synthesis process and (2) the requirement of poultry for ascorbic acid is high in case of hot weather or stressful condition [3]. The role of ascorbic acid has been demonstrated in previous studies. First, ascorbic acid is added as an additional nutrient to the diet to improve poultry performance by improving body weight and reducing mortality [4]. Furthermore, the use of ascorbic acid was aimed at improving the immune response and antioxidant capacity of birds [5-8]. In particular, in a heat stress environment, ascorbic acid had contributed to the energy supply of poultry birds by corticosterone biosynthesis [5]. In addition, the use of ascorbic acid also plays a crucial role in the treatment and prevention of *Salmonella enteritidis* [9]. Modulating physiological functions have also been recorded in some previous studies [6,7]. According to Gan *et al*. [10], ascorbic acid supplementation increased spleen ascorbic acid level and serum immunoglobulin G (IgG) levels.

**Table-1:** The characteristics of ascorbic acid substance [1].

Criteria	Ascorbic Acid (Vitamin C)
Formula based on empirical evidence	C_6_H_8_O_6_
The weight of a molecule	176.12 g/mol
Appearance	Crystals or powder ranging in color from white to slightly yellow.
Chemical abstract service number	50-81-7
PKa	4.7 (10°C)
Density	1.65 g/cm3
The point of melting	190–192°C
Solubility in Water	400 g/L (40°C)
Reactivity	Use bases, metals, air, and light to carry out the reactions in aqueous solution.

The use of ascorbic acid to support poultry animals in adverse conditions, especially under heat stress conditions, is essential; it is one of the important processes during the rearing period. Furthermore, the use of ascorbic acid in the poultry’s diet helps improve poultry’s performance and health. This review aims to synthesize all the information of ascorbic acid in the poultry’s diet, thereby providing the general role of ascorbic acid for the poultry industry.

## Ascorbic Acid Sources

Ascorbic acid is synthesized from glucose and is soluble in water [11]. Most animals cannot synthesize ascorbic acid endogenously, but poultry animals can synthesize it due to the L-gulonolactone oxidase enzyme, which is available in the poultry’s body. This enzyme is available in the kidney tissue where L-gulono-g-lactone is converted into ascorbic acid by the L-gulonolactone oxidase enzyme [12]. In birds, ascorbic acid is produced in the kidney, while in mammals, it is produced in the liver [5]. Ascorbic acid can be found in natural plants or synthesized through industrial processes. For plants, ascorbic acid is found from rose hips and sea buckthorn as the richest resource [13]. Furthermore, other fruits such as guava, star fruit, black currant, strawberry, and kiwi also contained a high amount of ascorbic acid. As we know, citrus fruits such as orange, lime, grapefruit, and so on contain ascorbic acid. Thus, the ascorbic acid content of the latter is much less than the former. The amount of ascorbic acid per fruit is shown in Table-2 [14-24]. This table shows that guava, cashew apple, and sea buckthorn contain high ascorbic acid levels.

**Table-2:** The amount of ascorbic acid in some plants and fruits.

Name	Family name	Latin name	Ascorbic acid content	Reference
Kiwi	Actinidiaceae	*Actinidia deliciosa*	60–78 mg/100 g of fresh weight	[14]
Star fruit	Oxalidaceae	*Averrhoa carambola* L.	1626 mg/100 g of juice	[15]
Guava	Myrtaceae	*Psidium guajava* L.	89–980 mg/100 g of fresh weight	[16,17]
Cashew apple	Anacardiaceae	*Anacardium occidentale* L.	555 mg/100 g of fresh weight	[14]
Orange	Rutaceae	*Citrus* x *sinensis* (L.)	41–58 mg/100 g of fresh weight	[14,18]
Common mandarin	Rutaceae	*Citrus reticulata* Blanco	27 mg/100 g of fresh weight	[14]
Black currant	Grossulariaceae	*Ribes nigrum* L.	148–310 mg/100 g of fresh weight	[19]
Broccoli	Brassicaceae	*Brassica oleracea* var*. italica* Plenck.	25–130 mg/100 g of fresh weight	[20]
Kale	Brassicaceae	*Brassica oleracea* var*. acephala*	51–120 mg/100 g of fresh weight	[20]
Potato	Solanaceae	*Solanum tuberosum* L.	8–30 mg/100 g of fresh weight	[21]
Tomato	Solanaceae	*Solanum lycopersicum* L.	9–17 mg/100 g of fresh weight	[22]
Sea buckthorn	Eleagnaceae	*Hippophaё rhamnoides* L.	70–1320 mg/100 g of juice	[23]
Coriander	Apiaceae	*Coriandrum sativum* L.	48–98 mg/100 g of fresh weight	[24]
Chives	Amaryllidaceae	*Allium schoenoprasum* L.	93 mg/100 g of fresh weight	[24]
Parsley	Apiaceae	*Petroselinum crispum* (Mill.) Nym	59 mg/100 g of fresh weight	[24]

**Figure-1:**
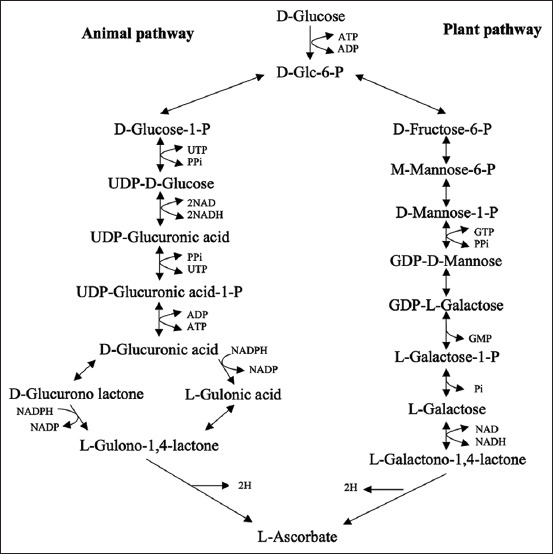
The pathway of ascorbic acid [26].

Due to a large number of sources of ascorbic acid, it has been widely used in the food processing industry to supplement the nutritional requirement of most food products. Ascorbic acid is the active ingredient responsible for many important biological processes. At present, there are two main processes for producing ascorbic acid, which were described in the study by Vandamme and Revuelta [25]. The adapted fermentation process and the Reichstein process are the most commonly used fermentation techniques in the industrial production of ascorbic acid. Both processes require some chemically based phases. According to the Reichstein method, D-glucose is catalytically hydrogenated to D-sorbitol and converted to L-sorbose by *Gluconobacter* spp. Thereafter, the L-sorbose is oxidized to produce 2-keto-L-gulonic acid. Lactonization is used to convert 2-keto-L-gulonic acid into L-ascorbic acid. In the two-step fermentation method, 2-keto-L-gulonic acid is produced from L-sorbose. It is then used in the second phase of production, which is replaced by bioconversion using different bacteria [25]. Ascorbic acid pathways are shown in Figure-1 [26].

## The Mechanism of Ascorbic Acid

Ascorbic acid is considered a powerful water-soluble antioxidant that neutralizes reactive oxygen species (ROS) and reduces oxidative stress in various studies [27,28]. Moreover, when it comes to biological systems, ascorbic acid is a powerful reducing agent and a free radical scavenger [29]. It serves as the body’s first line of defense against free radicals, and it also protects proteins and lipid membranes from oxidative stress. As a water-soluble compound, ascorbic acid plays a role in body cells’ interior and exterior parts, where it neutralizes free radicals and prevents free radical damage. In addition, ascorbic acid is an excellent source of electrons for free radicals, which are always looking for an electron to restore its stability. Ascorbic acid can donate electrons to free radicals, thereby reducing their reactivity [27]. Moreover, the activity of ascorbic acid is a result of the acid’s ability to act as an electron transfer agent (as a reducing agent) in the biological system [30]. This ability is considered a key enzyme cofactor, which normally helps to increase the enzyme activity by keeping the reduced forms of iron (Fe^2+^) and copper (Cu^+^) at the active site of the enzyme [31].

Although this cofactor has been recorded for many enzymatic functions, it is perhaps best known for its role in collagen protein production [31]. In addition to increasing iron absorption under normal conditions, ascorbic acid increases iron absorption from non-heme iron sources by reducing Fe^3+^ to Fe^2+^ [32]. When redox-active ions are present in the environment, ascorbic acid functions as a pro-oxidant contributing to the formation of hydroxyl radicals, which can result in lipid, DNA, or protein oxidation, among other things [27,28]. Due to its reducing capacity, ascorbic acid is an important biological antioxidant that protects against ROS that gains electrons from adjacent biological systems and other harmful substances [32]. In the events where ascorbic acid or other antioxidants are absent, ROS causes unwanted alterations to structures in biology such as DNA, RNA, proteins, and lipids. These modifications can cause mutations [30,32,33].

## Antioxidant Role of Ascorbic Acid in Poultry Production

The ability of ascorbic acid to transfer electrons allows it to have exceptional antioxidant qualities and helps maintain the integrity of various cells, including lymphocytes, by protecting them from damage caused by free radicals created in response to toxins or infection [34]. In poultry, the plasma antioxidant system is a vital aspect of the oxidation process that impacts the animal’s ability to cope with stress. The plasma antioxidant capacity is usually measured using markers such as superoxide dismutase (SOD), total antioxidant capacity (T-AOC), and glutathione peroxidase activities, as well as malondialdehyde (MDA) content [35,36]. Table-3 [6,36-39] shows some important markers of antioxidant capacity in poultry when supplemented with ascorbic acid in the diet. To keep host cells safe from free radicals and ROS, ascorbic acid, an excellent antioxidant, is essential in the diet [40,41] because ROS produced in excess due to infection might be detrimental to it [42]. Furthermore, it is more vulnerable when animals are older because ROS is produced more in the body.

**Table-3:** The effect of ascorbic acid on antioxidant defense system in poultry production.

AA amount	Breed	Criteria	Treatment		Bird’s age	Reference

			Ctr	Add		
12 mg/egg AA *in ovo* injection	Ross 708 breeder hens	MDA (nmol/mL)↓	6.49 7.90	4.19 5.05	At 27 days At 40 days	[36]
3 mg/egg AA *in ovo* injection	Fertile Chinese yellow broiler	MDA (nmol/mL)↓ GHS-Px (U/mL)↑ T-AOC (U/mL)↑	2.0 250 1.8	1.5 280 5.0	At 18 days	[6]
3 mg/egg AA *in ovo* injection	Arbor Acres broiler	T-AOC (U/mL)↑	5.43	7.03	At 42 days	[37]
Vitamin C 500mg/kg feed	White Pekin ducks	SOD (U/gHb)↑	37.4 32.6	66.4 71.8	At 23 days At 46 days	[38]
800 mg vitamin C/kg feed	Jin-ding female layer ducks	MDA (nmol/ml)↓	8.01 7.12	3.25 3.91	At 5 days At 28 days	[39]

↓: Decreased; ↑: Increased; AA: Ascorbic acid

For daily feeding, poultry requires more ascorbic acid in their diet than any other animal, first for nutritional requirements and then second to combat oxidative stress. Thus, the amount of ascorbic acid needed still depends on poultry bird, environment, state of oxidative stress, etc. From Table-3, it was observed that when ascorbic acid is added to the diet, it helps decrease the MDA, while it also helps increase T-AOC, GHS-Px, and SOD. It follows a growth phase one by one. The content of T-AOC and GSH was enhanced in the liver of aged egg-laying chickens when 0.25 g ascorbic acid/kg feed was added to the diet [10]. A high dose of ascorbic acid supplementation with more than 1 g/kg feed has shown to have deleterious effects on the release of the enzymes of an endogenous antioxidant such as GSH and T-AOC.

However, the increasing tendency of T-AOC and GSH levels in the liver of aged laying hens after dietary supplementation with ascorbic acid indicates that these chickens have a stronger antioxidant defense. Wang *et al*. [39] found that adding ascorbic acid in duck feed significantly reduced oxidizing agents in ducks. According to the previous studies, the increased antioxidant capacity of the old laying hens may have reduced their susceptibility, damage, and chance of mortality. However, Jena *et al*. [43] found that dietary supplementation of ascorbic acid increases blood antioxidant T-AOC. El-Senousey *et al*. [2] found that dietary supplementation with ascorbic acid can help reduce the production of inflammatory cytokines in the body in response to oxidative stress. The integration of ascorbic acid supplementation into broiler chicken’s feed exposed to oxidative stress from dexamethasone resulted in significant reductions in the levels of mRNA expression of interleukin-1b (IL-1b), interleukin-6 (IL-6), and interferon-g (IFNg) in the spleen of poultry [2]. It can be concluded that ascorbic acid supplementation can improve the performance of laying hens by increasing their antioxidant activity.

## Immunomodulation Role of Ascorbic Acid in Poultry Production

Due to the high costs of treating the disease and possible adverse health consequences, preventive medicine is always preferred over curative medicine [44]. Producing poultry with a competent immune system is an important goal in poultry production because the immune system plays an important role in disease prevention and high performance in poultry production. The innate response among other things helps prevent the spread of disease and leads to a more robust response to immunization. In poultry, the spleen is the largest immune organ and plays an important role in cellular and humoral immunity because birds lack lymphatic arteries and lymph nodes; the spleen plays a more major role in the immune function than the immune systems in other animals. Phagocytosis of damaged cells and antigens and the formation, storage, and maturation of lymphocytes are the two primary mechanisms by which the immune system of adult birds protects them from infection [45].

When ascorbic acid is added to the diet, chickens can be expected to see an improvement in their immune system due to its ability to lower the levels of adrenocorticotropic hormone. Immune and cytotoxicity suppressions are both effects of adrenocorticotropic hormone [46]. In addition, ascorbic acid increases the activity of the hexose monophosphate pathway, increasing the development of lymphoid cells, and thus, the production of antibodies increases [47]. A possible explanation for the decrease in ascorbic acid levels after chickens are exposed to infectious diseases is an increase in ROS generation due to the immune response against the pathogens [48,49]. Various factors influence immune system function. Of all vitamins, ascorbic acid provides one of the most significant effects. The previous studies have shown that ascorbic acid can increase plasma levels of IgG and immunoglobulin M (IgM), as well as the activity of the lysozyme enzyme [50]. Further, research revealed that the gene regulatory actions of ascorbic acid may play a role in the immunomodulatory function of lymphocytes by regulating the expression of immunoglobulins [51].

Zhu *et al*. [37] found that the addition of 3 mg ascorbic acid (*in ovo*) increased IgG on the 21^st^ day and IgM on both the 1^st^ and the 21^st^ days. Specifically, the IgG level was 5.410 g/L in the treatment group compared to the control group, which is 3.234 g/L. Moreover, the IgM in the treatment group is 4.939 g/L compared to 2.974 g/L in the control group. Different studies have shown that ascorbic acid can increase the amount of IgG and IgM in the serum and the activity of the enzyme lysozyme [50]. However, while the role of ascorbic acid in lymphocytes is less, research has revealed that the gene regulatory effect of ascorbic acid may play a role in the immunomodulatory function of lymphocytes, by regulating the expression of immunoglobulins [51]. According to Gan *et al*. [10], increased concentrations of ascorbic acid and DHA in the spleen due to dietary supplementation of ascorbic acid may be a contributing factor to the increased CD4^+^ T lymphocyte numbers and IgG concentrations of 78-week-old laying hens. B cells can differentiate to produce IgM and it has been shown that ascorbic acid can facilitate this process. Due to the increased numbers of CD4^+^ T lymphocytes, the B cells may become more active. In addition, ascorbic acid can increase antibody formation as part of the immune response to infection [52].

Supplementation with 200 mg/kg of ascorbic acid in feed resulted in increased antibodies preventing Newcastle disease virus (NDV) and decreased the lymphocytes in poultry [53]. Increased humoral and cellular immunity has been reported in the pigeons fed diets containing 300 mg ascorbic acid per kg feed [54]. Bendich [55] reported that the availability of ascorbic acid is required for the systemic presence of IFNs and immunoglobulins. Apart from that, ascorbic acid supplements improved blood CD8^+^ and IgM levels. Different doses of ascorbic acid supplemented with 50-200 ppm in feeds have found different effects on post-vaccination antibodies that protect the body from NDV and infectious bursal disease virus (IBDV) [56]. In particular, birds that received 200 ppm of ascorbic acid had significantly higher antibodies against IBDV than birds that received less. This revealed that ascorbic acid has good effects in improving antibody-mediated immunity in broilers.

According to Gan *et al*. [10], the effects of ascorbic acid supplementation in laying hen’s diet showed promising results with more ascorbic acid in the animal’s system. The addition of ascorbic acid drastically reduced the ability to synthesize L-gluconolactone oxidase, demonstrating the availability of an internal feedback system in poultry animals that managed the ascorbic acid production. Birds provided a diet containing 200 mg/kg ascorbic acid showed that IFN-g, IL-6, and IL-1b of mRNA expression levels in the spleens were reduced [2]. Therefore, it can be inferred that the addition of ascorbic acid in poultry diets strengthens immunity and improves health and performance.

## The Interaction of Ascorbic Acid with Vitamin E and Other Substances

Ascorbic acid and vitamin E have a strong relationship and have beneficial effects on the immune system through increasing macrophage activity in birds, antibody synthesis, and humoral immunity in poultry. When combined with vitamin E, ascorbic acid acts as an antioxidant or pro-oxidant, helping it maintain vitamin E levels by reducing the degraded metabolism of vitamin E’s and thereby increasing its antioxidant efficiency [57]. As a result, the combination of these two substances can be effective in improving the performance and immunological response of poultry. Shakeri *et al*. [57] also discussed that vitamin E and ascorbic acid work together to protect lipids and proteins from oxidative stress, making a combination of the two a viable options for treating chronic heat stress. vitamin E and ascorbic acid play a crucial role in oxygen radical scavenging and helping the immune system to function better. Ascorbic acid also reproduces vitamin E by limiting the formation of vitamin E radicals. According to Hashem *et al*. [58], co-supplementation of ascorbic acid and vitamin E, either alone or in combination, improved hematological and biochemical markers in the diets with CuSO_4_-induced toxicity. In addition, ascorbic acid and vitamin E supplementation reduced oxidative stress and histological changes in the kidney. Moreover, supplementation of ascorbic acid with probiotics and vitamin E effectively reduces the negative impact of chronic heat stress on growth performance and immunity of poultry [59].

Compared with broiler poultry, layer poultry fed a diet enriched with zinc and ascorbic acid had increased weights of the thymus, bursa, and spleen [54]. The combination of ascorbic acid and other dietary supplements reduced oxidative stress *in vivo* and poultry’s diet. In the Ross 308 broiler’s diet, the combination of vitamin E, ascorbic acid, and selenium treatment was superior to individual supplementation in terms of oxidative stress induced by high n-3 dietary polyunsaturated fatty acid intake [60]. Moreover, Amer *et al*. [61] found that ascorbic acid combined with dietary safflower oil improved the apparent ileal digestibility of ascorbic acid and ileum. In the case of combining 200 mg/kg ascorbic acid and 1.5 mg/kg folic acid, Gouda *et al*. [53] showed that Cobb 500 broilers grown under heat stress showed better growth performance, antioxidant enzyme activity, serum biochemical indicators, and enhanced immune function. In the study of Rajabi and Torbi [62], the integration of ascorbic acid and zinc sulfate improved the Haugh unit and shell thickness. Therefore, the study suggested that adding 240 mg/kg of ascorbic acid in feed was ideal for laying poultry. In addition, the inclusion of ascorbic acid and fiber in the diet will not only improve poultry performance but also improve poultry’s skeletal structure. The addition of ascorbic acid also improved the wing and tail feather integrity with a lower feed allowance.

## Heat Stress Management

### Effect of heat stress on poultry performance

**Figure-2:**
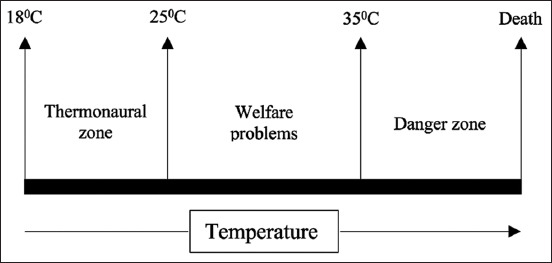
The temperature between zones in poultry production [66].

In addition to inadequate ventilation, high stocking densities, and human interaction, modern chicken production is often accompanied by additional stressors that can increase the stress associated with a high-temperature environment. Heat stress occurs when poultry cannot dissipate heat beyond their body temperature into the surrounding environment, causing the bird to become dehydrated. Poultry cannot sweat, and their body feathers significantly inhibit their ability to release heat into the intermediate environment [63]. Based on the facts presented above, it is recognized that heat stress can cause significant harm to organisms. It is possible to experience biochemical and physiological changes, such as the emergence of the stress hormone, increased free radical generation, and lower antioxidant status and decreased resistance change in homeostasis [63]. Emphasis should be noted that heat stress is one of the most common factors that can affect meat quality in the environment [64,65]. High temperature in the surrounding environment can reduce performance and increase mortality. At present, heat stress is a common concern, particularly in poultry production systems, during the summer or dry season. Heat stress is affected by the temperature of the surrounding environment and the amount of heat exchange available to the animal due to the humidity ambient, ventilation, and animal density [8]. In other words, when the external temperature is higher than the poultry’s optimal temperature for birds, their body temperature results in heat stress with a combination of many factors in the bird’s environment [64]. Figure-2 shows the temperature stages in poultry production [66].

When 18-25°C is used as the reference temperature, the feed intake of broilers or laying poultry decreases with each degree increase in temperature. Furthermore, many environmental parameters such as thermal irradiation, sunlight, air humidity, and animal characteristic such as metabolic rate, thermoregulatory mechanisms, and species-predilection all contribute to the imbalance in heat production in birds [67]. Poultry has a normal body temperature of 41-42°C, and their thermal comfort zone is 18-25°C [68]. Temperatures above 25°C cause heat stress in chickens, according to a study by Wasti *et al*. [69]. Heat stress can result in decreased feed intake and various physiological responses [68], and all these can adversely affect chicken growth performance. Chick temperatures should be set to about 35°C for the 1^st^ week after hatching and then reduced by 3°C weekly until a typical ambient temperature of around 23-26°C is reached [68]. Broiler meat and eggs lose quality at high temperatures, causing postharvest losses. When broiler chicks are exposed to high ambient temperatures during the growth phase, Ranjan *et al*. [70] found that poor meat quality and storage quality problems will persist.

## Alleviating Heat Stress Role in Poultry Production

Some researchers [71,72] have experimented with dietary ascorbic acid to improve poultry performance to combat the detrimental effect of heat stress on poultry production. Ascorbic supplementation in poultry feeds has beneficial effects under heat stress conditions, and this includes increased weight gain, enhanced immune response, and other effects on their performance. Some results suggest that supplementing ascorbic acid in the diet can improve antibody-mediated responses under heat stress conditions in poultry. Panda *et al*. [71] produced a significant result for immunomodulatory effects of ascorbic acid, 44-45 weeks on white leghorn chickens treated with ascorbic acid.

Similarly, Shin *et al*. [72] noted that the addition of ascorbic acid had been shown to protect meat ducks during heat stress conditions. In times of stress, ascorbic acid is essential for the production process of corticosterone hormone, which increases the availability of energy to the body [5]. Corticosteroid secretion increases in response to stress at temperatures above or below the neutral heat zone (18-22°C). Kumar *et al*. [73] showed that in reaction to stress, ascorbic acid reduces or alleviates the negative effects of stress in poultry performance by reducing the secretion and synthesis of corticosteroids. Consequently, the mortality rate during heat stress is generally reduced with adequate ascorbic acid delivery.

Under heat stress conditions, birds cannot synthesize adequate levels of ascorbic acid in the body. The supplementation of ascorbic acid has been shown to reduce the temperature of birds significantly. According to Abidin and Katoon [74], ascorbic acid supplementation alleviates the problem caused by heat stress in birds and improves performance such as increased weight gain, better feed intake, increased carcass weight, and decreased mortality. Body temperatures, immunity state, and oxidative stress were also improved. Dietary treatment with ascorbic acid reduces and decreases stress-induced metabolic indicators. It also improved performance and decreased mortality and immunological status, among other benefits. According to the above study results, ascorbic acid supplementation of 250 mg/kg proved to provide the optimal response in terms of growth performance, feed efficiency, feed conversion ratio, carcass quality, and survival rate in broiler poultry under heat stress [75]. However, the amount of ascorbic acid required in layers, such as shell/egg fracture ratio under heat stress, is approximately 200-500 mg/kg of body weight to achieve maximum egg production, feed efficiency, and egg quality [74,76]. In addition, some studies have suggested different amounts of ascorbic acid on poultry performance, as shown in Table-4 [8,38,77-82].

**Table-4:** The influences of ascorbic acid on poultry performance under heat stress condition.

Breed	Age	AA	Temperature	Performance	Reference
Commercial L_33_ layer hens	39 weeks	150 mg ascorbic acid per kg diet	35.9°C in 24 hours/day	- Increased eggs weight, egg quality	[77]
Commercial broiler birds	96 days old	200 mg of ascorbic acid in diet	37±5.0°C in 45 days	- Increased in plasma glucose concentration	[78]
				- Reduction in thiobarbituric acid value	
Male pekin ducks	1-day old	300 mg/kg ascorbic acid in water (plus 800 mg/kg betaine)	11:00–17:00 h, 33–40°C	- Increase body weight, feed efficiency	[79]
				- Increase in blood cells, hemoglobin, …	
				- Reduction in gene expression of heat shock protein 70 (HSP70) in the liver	
Male Ross 308 broiler chicks	One-day-old	The amount of 200 g vitamin C per 1,000 litters in drinking water	35°C from 08:00 to 13:00 h each day	- Final body weight, and average weight gain were increased while feed conversion ratio was decrease	[8]
				- Reduced corticosterone concentration	
Fertile broiler eggs from Cobb 500	Eggs and chicks from 1-42 days	6 mg/100 mL ascorbic acid injection	32°C for entire experiment	- Diminishing and increasing the muscle fiber area to prevent from hot temperature	[80]
Isa Brown laying hens	13 months	One kilogram per one ton feed	23.84 Celsius degrees and 25.54 Celsius degrees	- No difference in egg production between different temperature	[81]
White Pekin ducks	twelve weeks old	250 mg or 500 mg per kg of feed	Maximum temperature was 39.16°C	- Improved the haemato-biochemical and oxidative parameters	[38]
Cobb 500 broilers	One-day-old	the amount of 2 g/L drinking water	38±1°C	- Decreased feed conversion ratio. Increased the volume of carcass, legs, thighs, and back	[82]

## Conclusion

As a water-soluble substance, ascorbic acid can be conveniently added to drinking water and feed diets of the poultry. The application of ascorbic acid on poultry has brought many benefits to the poultry industry. Ascorbic acid has been found to improve poultry performance, especially in heat stress conditions. The immune system was also improved with the addition of ascorbic acid to the diet. In particular, ascorbic acid improves the responses to infection and inflammations. To increase the overall growth of birds, ascorbic acid can be added to the ratio with the addition of other substances such as vitamin E, zinc, safflower oil, folic acid, and fibrous diet. The amount of ascorbic acid depends on the targeted poultry species and the purpose of poultry farming. The effect of ascorbic acid will vary from low to high depending on poultry breed, diet, and ambient temperature. Further studies should be more focused on the application of ascorbic acid as alternative to antibiotics.

## Authors’ Contributions

TVH and NTKQ: Conceptualized and designed the review. NHQ and FAAH: Drafted the manuscript. BG: Revised the manuscript. All authors read and approved the final manuscript.

## References

[1] (2021). National Center for Biotechnology Information.

[2] El-Senousey H.K, Chen B, Wang J.Y, Atta A.M, Mohamed F.R, Nie Q.H (2018). Effects of dietary vitamin C, vitamin E, and alpha-lipoic acid supplementation on the antioxidant defense system and immune-related gene expression in broilers exposed to oxidative stress by dexamethasone. Poul. Sci.

[3] Whitehead C.C, Keller T (2003). An update on ascorbic acid in poultry. Worlds Poul. Sci. J.

[4] Shewita R.S, El-Naggar K, El-Naby W.S.A (2019). Influence of dietary vitamin C on growth performance, blood biochemical parameters and transcript levels of heat shock proteins in high stocking density reared broiler chickens. Slov. Vet. Res.

[5] Ahmadu S, Mohammed A.A, Buhari H, Auwal A (2016). An overview of vitamin C as an antistress in poultry. Malays. J. Vet. Res.

[6] El-Senousey H.K, Chen B, Wang J.Y, Atta A.M, Mohamed F.R, Nie Q.H (2018). *In ovo* injection of ascorbic acid modulates antioxidant defense system and immune gene expression in newly hatched local Chinese yellow broiler chicks. Poul. Sci.

[7] Min Y.N, Niu Z.Y, Sun T.T, Wang Z.P, Jiao P.X, Zi B.B, Chen P.P, Tian D.L, Liu F.Z (2018). Vitamin E and vitamin C supplementation improve antioxidant status and immune function in oxidative stressed breeder roosters by up-regulating expression of GSH-Px gene. Poult. Sci.

[8] Barrio A.S, Mansilla W.D, Navarro-Villa A, Mica J.H, Smeets J.H, den Hartog L.A, García-Ruiz A.I (2020). Effect of mineral and vitamin C mix on growth performance and blood corticosterone concentrations in heat-stressed broilers. J. Appl. Poul. Res.

[9] Hernandez-Patlan D, Solis-Cruz B, Pontin K.P, Latorre J.D, Hernandez-Velasco X, Merino-Guzman R, Mendez-Albores A, Hargis B.M, Lopez-Arellano R, Tellez-Isaias G (2019). Evaluation of ascorbic acid or curcumin formulated in a Solid Dispersion on *Salmonella enteritidis* infection and intestinal integrity in broiler chickens. Pathogens.

[10] Gan L, Fan H, Nie W, Guo Y (2018). Ascorbic acid synthesis and transportation capacity in old laying hens and the effects of dietary supplementation with ascorbic acid. J. Anim. Sci. Biotechnol.

[11] Sahin K, Onderci M, Sahin N, Gursu M.F, Kucuk O (2003). Dietary vitamin C and folic acid supplementation ameliorates the detrimental effects of heat stress in Japanese quail. J. Nutr.

[12] Hooper C.L, Maurice D.V, Lightsey F, Toler E (2001). Factors affecting ascorbic acid biosynthesis in chickens I. Adaptation of an assay and the effect of age, sex and food deprivation. J. Anim. Physiol. Anim. Nutr.

[13] Roman I, Stănilă A, Stănilă S (2013). Bioactive compounds and antioxidant activity of *Rosa canina* L*.* biotypes from spontaneous flora of Transylvania. Chem. Cent. J.

[14] Ellong E, Billard C, Adenet S, Rochefort K (2015). Polyphenols, carotenoids, vitamin C content in tropical fruits and vegetables and impact of processing methods. Food Sci. Nutr.

[15] Ariharan V.N, Kalirajan K, Devi V.N, Prasad P (2012). An exotic fruit which forms the new natural source for vitamin C. Rasayan J. Chem.

[16] Gull J, Sultana B, Anwar F, Naseer R, Ashraf M, Ashrafuzzaman M (2012). Variation in antioxidant attributes at three ripening stages of guava (*Psidium guajava* L.) fruit from different geographical regions of Pakistan. Molecules.

[17] McCook-Russell K.P, Nair M.G, Facey P.C, Bowen-Forbes C.S (2012). Nutritional and nutraceutical comparison of Jamaican *Psidium cattleianum* (strawberry guava) and *Psidium guajava* (common guava) fruits. Food Chem.

[18] Najwa R, Azlan A (2017). Comparison of vitamin C content in citrus fruits by titration and high-performance liquid chromatography (HPLC) methods. Int. Food Res. J.

[19] Vagiri M, Ekholm A, Öberg E, Johansson E, Andersson S.C, Rumpunen K (2013). Phenols and ascorbic acid in black currants (*Ribes nigrum* L.):Variation due to genotype, location, and year. J. Agric. Food Chem.

[20] Domínguez-Perles R, Mena P, García-Viguera C, Moreno D.A (2014). Brassica foods as a dietary source of vitamin C:A review. Crit. Rev. Food Sci. Nutr.

[21] Kulen O, Stushnoff C, Holm D.G (2013). Effect of cold storage on total phenolic content, antioxidant activity and vitamin C level of selected potato clones. J. Sci. Food Agric.

[22] Georgé S, Tourniaire F, Gautier H, Goupy P, Rock E, Caris-Veyrat C (2011). Changes in the contents of carotenoids, phenolic compounds and vitamin C during technical processing and lyophilisation of red and yellow tomatoes. Food Chem.

[23] Gutzeit D, Baleanu G, Winterhalter P, Jerz G (2008). Vitamin C content in sea buckthorn berries (*Hippophaërhamnoides* L ssp.* rhamnoides*) and related products:A kinetic study on storage stability and the determination of processing effects. J. Food Sci.

[24] Santos J, Herrero M, Mendiola J, Oliva-Teles M.T, Ibáñez E, Delerue-Matos C, Oliveira M (2014). Fresh-cut aromatic herbs:Nutritional quality stability during shelf-life. LWT.

[25] Vandamme E.J, Revuelta J.L (2016). Industrial fermentation of vitamin C. In:Industrial Biotechnology of Vitamins, Biopigments, and Antioxidants.

[26] Hancock R.D, Viola R (2002). Biotechnological approaches for L-ascorbic acid production. Trends Biotechnol.

[27] Rouhier N, Lemaire S.D, Jacquot J.P (2008). The role of glutathione in photosynthetic organisms:Emerging functions for glutaredoxins and glutathionylation. Annu. Rev. Plant Biol.

[28] Verma R.S, Mhta A, Srivastava N (2007). *In vivo* chlorpyrifos induced oxidative stress:Attenuation by antioxidant vitamins. Pestic. Biochem. Phys.

[29] Duarte T.L, Lunec J (2005). Review:When is an antioxidant not an antioxidant?A review of novel actions and reactions of vitamin C. Free Radic. Res.

[30] Timberlake K.C (2015). General, Organic, and Biological Chemistry:Structures of Life.

[31] Davey M.W, van Montagu M, Inze D, Sanmartin M, Kanellis A, Smirnoff N, Benzie I.J.J, Strain J.J, Favell D, Fletcher J (2000). Plant L-ascorbic acid:Chemistry, function, metabolism, bioavailability and effects of processing. J. Sci. Food Agric.

[32] Carocho M, Ferreira I.C.F (2013). A review on antioxidants, pro-oxidants and related controversy:Natural and synthetic compounds, screening and analysis methodologies and future perspectives. Food Chem. Toxicol.

[33] Craft B.D, Kerrihard A.L, Amarowicz R, Pegg R.B (2012). Phenol-based antioxidants and the *in vitro* methods used for their assessment. Comprehensive Rev. Food Sci. Food Saf.

[34] Nimse S.B, Pal D (2015). Free radicals, natural antioxidants, and their reaction mechanisms. RSC Adv.

[35] Yang X, Li L, Duan Y.L, Yang X (2017). Antioxidant activity of *Lactobacillus plantarum* JM113 *in vitro* and its protective effect on broiler chickens challenged with deoxynivalenol. J. Anim. Sci.

[36] Zhang H, Elliott K.E.C, Durojaye O.A, Fatemi S.A, Schilling M.W, Peebles E.D (2019). Effects of *in ovo* injection of L-ascorbic acid on growth performance, carcass composition, plasma antioxidant capacity, and meat quality in broiler chickens 1, 2, 3. Poult. Sci.

[37] Zhu Y.F, Li S.Z, Sun Q.Z, Yang XJ (2019). Effect of *in ovo* feeding of vitamin C on antioxidation and immune function of broiler chickens. Animals.

[38] Behera H, Jena G, Kumar D, Mishra S, Das D, Samal L, Dalai L (2020). Ameliorative effect of vitamin C on hemato-biochemical and oxidative parameters in ducks during summer. Int. J. Livestock Res.

[39] Wang A, Xie F, Wang Y.H, Wu J.L (2011). Effects of vitamin C supplementation on growth performance and antioxidant status of layer ducklings. J. Anim. Physiol. Anim. Nutr.

[40] Fukumura H, Sato M, Kezuka K, Sato I, Feng X, Okumura S, Fujita T, Yokoyama U, Eguchi H, Ishikawa Y, Saito T (2012). Effect of ascorbic acid on reactive oxygen species production in chemotherapy and hyperthermia in prostate cancer cells. J. Physiol. Sci.

[41] Barrita J.L.S, Sanchez M.S.S (2013). Antioxidant role of ascorbic acid and its protective effects on chronic diseases. In:Oxidative Stress and Chronic Degenerative Diseases-a Role for Antioxidants. InTech, Rijeca.

[42] Kohchi C, Inagawa H, Nishizawa T, Soma G.I (2009). ROS and innate immunity. Anticancer Res.

[43] Jena B.P, Panda N, Patra R.C, Mishra P.K, Behura N.C, Panigrahi B (2013). Supplementation of vitamin E and C reduces oxidative stress in broiler breeder hens during summer. Food Nutr. Sci.

[44] Rheinberger C.M, Herrera-Araujo D, Hammitt J.K (2016). The value of disease prevention vs treatment. J. Health Econ.

[45] Smith K.G, Hunt J.L (2014). On the use of spleen mass as a measure of avian immune system strength. Oecologia.

[46] Pardue S.L, Thaxton J.P, Brake J (1985). Role of ascorbic acid in chicks exposed to high environmental temperature. J. Appl. Physiol.

[47] Dieter M.P, Breitenbach R.P (1971). Vitamin C in lymphoid organs of rats and cockerels treated with corticosterone or testosterone. Proc.Soc. Exp. Biol. Med.

[48] Kawashima A, Sekizawa A, Koide K, Hasegawa J, Satoh K, Arakaki T, Takenaka S, Matsuoka R (2015). Vitamin C induces the reduction of oxidative stress and paradoxically stimulates the apoptotic gene expression in extravillous trophoblasts derived from first-trimester tissue. Reprod. Sci.

[49] Zhong X, Zeng M, Bian H, Zhong C, Xiao F (2017). An evaluation of the protective role of vitamin C in reactive oxygen species-induced hepatotoxicity due to hexavalent chromium *in vitro* and *in vivo*. J. Occup. Med. Toxicol.

[50] Yao B.B (2014). On serum immunoglobulin A and G influenced by the intake of vitamin C after aerobic exercise exhaustion:A case study of martial arts. J. Shijiazhuang Univ.

[51] Carr A.C, Maggini S (2017). Vitamin C and immune function. Nutrients.

[52] Ichiyama K, Mitsuzumi H, Zhong M, Tai A, Tsuchioka A, Kawai S, Yamamoto I, Gohda E (2009). Promotion of IL-4-and IL-5-dependent differentiation of anti-mu-primed B cells by ascorbic acid 2-glucoside. Immunol. Lett.

[53] Gouda A, Amer S.A, Gabr S, Tolba S.A (2020). Effect of dietary supplemental ascorbic acid and folic acid on the growth performance, redox status, and immune status of broiler chickens under heat stress. Trop. Anim. Health Prod.

[54] Chand N, Naz S, Khan A, Khan S, Khan R.U (2014). Performance traits and immune response of broiler chicks treated with zinc and ascorbic acid supplementation during cyclic heat stress. Int. J. Biometeorol.

[55] Bendich A (1990). Antioxidant vitamins and their functions in immune responses. In:Antioxidant Nutrients and Immune Functions. Vol. 262. Springer, Berlin, Germany.

[56] Lohakare J.D, Ryu M.H, Hahn T.W, Lee J.K, Chae BJ (2005). Effects of supplemental ascorbic acid on the performance and immunity of commercial broilers. J. Appl. Poult. Res.

[57] Shakeri M, Oskoueian E, Le H.H, Shakeri M (2020). Strategies to combat heat stress in broiler chickens:Unveiling the roles of selenium, vitamin E and vitamin C. Vet. Sci.

[58] Hashem M.A, Abd El Hamied S.S, Ahmed E.M.A, Amer S.A, Hassan A.M (2021). Alleviating effects of vitamins C and E supplementation on oxidative stress, hematobiochemical, and histopathological alterations caused by copper toxicity in broiler chickens. Animals.

[59] Attia Y.A, Al-Harthi M.A, El-Shafey A.S, Rehab Y.A, Kim W.K (2017). Enhancing tolerance of broiler chickens to heat stress by supplementation with vitamin E, vitamin C and/or probiotics. Ann. Anim. Sci.

[60] Leskovec J, Levart A, Svete A.N, Peric L, Stojcic M.Ð, Z?ikic D, Salobir J, Rezar V (2018). Effects of supplementation with a-tocopherol, ascorbic acid, selenium, or their combination in linseed oil-enriched diets on the oxidative status in broilers. Poult. Sci.

[61] Amer S.A, Mohamed W.A, Gharib H.S, Al-Gabri N.A, Gouda A, Elabbasy M.T, Abd El-Rahman G.I, Omar A.E (2021). Changes in the growth, ileal digestibility, intestinal histology, behavior, fatty acid composition of the breast muscles, and blood biochemical parameters of broiler chickens by dietary inclusion of safflower oil and vitamin C. BMC Vet. Res.

[62] Rajabi M, Torki M (2021). Effect of dietary supplemental vitamin C and zinc sulfate on productive performance, egg quality traits and blood parameters of laying hens reared under cold stress condition. J. Appl. Anim. Res.

[63] Akbarian A, Michiels J, Degroote J, Majdeddin M, Golian A, DeSmet S (2016). Association between heat stress and oxidative stress in poultry;mitochondrial dysfunction and dietary interventions with phytochemicals. J. Anim. Sci. Biotechnol.

[64] Lara L.J, Rostagno M.H (2013). Impact of heat stress on poultry production. Animals.

[65] Wang Y, Zhao H, Shao Y, Liu J, Li J, Xing M (2017). Copper or/and arsenic induce oxidative stress-cascaded, nuclear factor kappa B-dependent inflammation and immune imbalance, trigging heat shock response in the kidney of chicken. Oncotarget.

[66] Youssef A, Exadaktylos V, Berckmans D.A (2015). Towards real-time control of chicken activity in a ventilated chamber. Biosyst. Eng.

[67] Rostagno M.H (2020). Effects of heat stress on the gut health of poultry. J. Anim. Sci.

[68] Gicheha M.G (2021). The effects of heat stress on production, reproduction, health in chicken and its dietary amelioration. In:Advances in Poultry Nutrition Research, Amlan Kumar Patra. IntechOpen, London.

[69] Wasti S, Sah N, Mishra B (2020). Impact of heat stress on poultry health and performances, and potential mitigation strategies. Animals.

[70] Ranjan A, Sinha R, Devi I, Rahim A, Tiwari S (2019). Effect of heat stress on poultry production and their management approaches. Int. J. Curr. Microbiol. Appl. Sci.

[71] Panda A.K, Ramarao S.V, Raju M.V, Chatterjee R.N (2008). Effect of dietary supplementation with vitamins E and C on production performance, immune responses and antioxidant status of white leghorn layers under tropical summer conditions. Br. Poult. Sci.

[72] Shin J.S, Um K.H, Park J.K, Choi Y.S, Lee H.S, Park B.S (2019). Effect of betaine and ascorbic acid in drinking water on growth performance and blood biomarkers in meat ducks exposed to heat stress. S Afr. J. Anim. Sci.

[73] Kumar A, Roy B, Ganguly S, Praveen P.K, Shekhar S, Dalai N (2014). Supplementation of vitamin C for health promotion and combating heat stress in poultry. Int. J. Biopharm. Res.

[74] Abidin Z, Khatoon A (2013). Heat stress in poultry and the beneficial effects of ascorbic acid (vitamin C) supplementation during periods of heat stress. Worlds Poult. Sci. J.

[75] Attia Y.A, Hassan R.A, Qota E.M.A (2009). Recovery from adverse effects of heat stress on slow-growing chicks in the tropics 1:Effect of ascorbic acid and different levels of betaine. Trop. Anim. Health Prod.

[76] Ali M, Howlider M, Azad A, Rahman M (2010). Vitamin C and electrolyte supplementation to support growth and meat yield of broilers in a hot humid environment. J. Bangladesh Agric. Univ.

[77] Ajakaiye J.J, Perez-Bello A, Mollineda-Trujillo A (2011). Impact of heat stress on egg quality in layer hens supplemented with l-ascorbic acid and dl-tocopherol acetate. Vet. Arh.

[78] Kumar K, Mishra A, Sheikh A.A, Patel P, Ahirwar M.K, Bashir S.M, Ali A (2017). Effect of ascorbic acid on some biochemical parameters during heat stress in commercial broilers. Int. J. Curr. Microbiol. Appl. Sci.

[79] Kumar S, Pangeni D, Yang X, Park S.O (2017). Effect of ascorbic acid in the drinking water and betaine in the diet on performance, blood haematology, IgG and HSP 70 gene expression in Pekin ducks (*Anas platyrhynchos domesticus*) reared under high temperatures. Eur. Poult. Sci.

[80] Ferreira I.B, Matos Junior J.B, Sgavioli S, Vicentini T.I, Morita V.S, Boleli I.C (2015). Vitamin C prevents the effects of high rearing temperatures on the quality of broiler thigh meat. Poult. Sci.

[81] Cilev G, Crnec I, Sefer D, Markovic R, Kochoski L, Stojanovski S, Pachinovski N (2020). The influence of vitamin C over the production performances of the laying hens in conditions of thermal stress. Biotech. Anim. Husbandry.

[82] Ružic Z, Kanački Z, Jokanovic M, Vidakovic S, Kneževic S, Jovic S, Paras S (2020). The influence of vitamin C and early-age thermal conditioning on the quality of meat and specific production characteristics of broilers during heat stress. Turk. J. Vet. Anim. Sci.

